# Thromboembolic Events and Role of Point of Care Ultrasound in Hospitalized Covid-19 Patients Needing Intensive Care Unit Admission

**DOI:** 10.1177/0885066620964392

**Published:** 2021-12

**Authors:** Sumit Kapoor, Sudham Chand, Vladyslav Dieiev, Melissa Fazzari, Tristan Tanner, David C. Lewandowski, Anil Nalla, Omar Abdulfattah, Michael S. Aboodi, Ariel L. Shiloh, Michelle N. Gong

**Affiliations:** 1 Division of Critical Care Medicine, Department of Internal Medicine, 2013 Montefiore Medical Center, Bronx, NY, USA; 2 Department of Epidemiology and Population Health, 2006 Albert Einstein College of Medicine, Bronx, NY, USA; 3 Division of Critical Care Medicine, Division of Pulmonary Medicine, Department of Internal Medicine, Montefiore Medical Center, Bronx, NY, USA

**Keywords:** Covid-19, thrombosis, ICU, POCUS, incidence, mortality

## Abstract

**Background:**

Covid-19 associated coagulopathy (CAC) is associated with prothrombotic state and thromboembolism. However, true incidence of thromboembolic events is difficult to determine in the ICU setting. The aim of our study was to investigate the cumulative incidence of thromboembolic events in Covid-19 patients needing intensive care unit (ICU) admission and assessing the utility of point of care ultrasound (POCUS) to screen for and diagnose lower extremity deep venous thrombosis (DVT).

**Methods:**

We conducted a prospective observational study between April 22nd and May 26th, 2020 where all adult patients with the diagnosis of Covid-19 pneumonia admitted to 8 ICUs of Montefiore Medical Center were included. POCUS exam was performed on all patients at day 1 of ICU admission and at day 7 and 14 after the first exam.

**Results:**

The primary outcome was to study the cumulative incidence of thromboembolic events in Covid-19 patients needing ICU admission. A total of 107 patients were included. All patients got POCUS exam on day 1 in the ICU, 62% got day 7 and 41% got day 14 exam. POCUS diagnosed 17 lower extremity DVTs on day 1, 3 new on day 7 and 1 new on day 14. Forty patients developed 52 thromboembolic events, with the rate of 37.3%. We found a high 45-day cumulative incidence of thromboembolic events of 37% and a high 45-day cumulative incidence of lower and upper extremity DVT of 21% and 10% respectively. Twelve (30%) patients had failure of therapeutic anticoagulation. Occurrence of a thromboembolic event was not associated with a higher risk of mortality (HR 1.08, p value = .81).

**Conclusions:**

Covid-19 patients in ICU have a high cumulative incidence of thromboembolic events, but not associated with higher mortality. POCUS is an excellent tool to help screen and diagnose DVT during a pandemic.

## Introduction

Coronavirus-19 induced disease (Covid-19) caused by novel SARS- CoV-2 virus, was declared a global pandemic by World Health Organization in March 2020, after the first case was diagnosed in Wuhan, China in December 2019.^1^ The disease affects lungs primarily, severity ranging from mild or asymptomatic cases to severe ones with acute respiratory distress syndrome (ARDS), septic shock and multi system organ failure.^2^ Covid-19 associated coagulopathy (CAC), a distinct form of coagulopathy, has been described in Covid-19 patients and is associated with higher morbidity and mortality.^3–8^ Patients with CAC develop prothrombotic state with significant elevation of d-dimer, fibrinogen, fibrin and fibrin degradation products, modest prolongation of PT and aPTT, normal or small decrease in platelet count and increased clot strength by thrombo-elastography.^6,9^ Multiple reports of venous thromboembolism (VTE), arterial thrombosis, acute stroke, clotting of vascular access catheters and dialysis circuits have been described in Covid-19 patients.^10–27^ Per published reports, the cumulative incidence of VTE (deep venous thrombosis and pulmonary embolism) in Covid-19 patients varies from 7.7% up to 69%.^10–12,16–27^ The pathophysiology of CAC includes 2 mechanisms: systemic inflammation or cytokine storm causing endothelial cell activation and injury in the lung micro vasculature leading to pulmonary micro thrombosis and a hypercoagulable state leading to large vessel thrombosis.^3,8,28^ Recent autopsy series of Covid-19 patients showed histologic pattern of diffuse alveolar damage, severe endothelial injury, widespread alveolar capillary thrombosis, micro angiopathy and angiogenesis.^29^ The true prevalence of thromboembolic complications in ICU patients with Covid-19 is difficult to estimate, as it may not be feasible to transport critically ill patients and there is a risk of nosocomial spread of the virus among healthcare staff and hospitalized patients, thereby limiting diagnostic imaging studies.^14^ It is also unclear if patients with Covid-19 associated coagulopathy are prone to develop thrombotic events with time and if thrombotic events are associated with higher mortality. The primary aim of our study was to prospectively investigate the cumulative incidence of thromboembolic events in hospitalized Covid-19 patients needing ICU admission and assessing the utility of point of care ultrasound (POCUS) to screen and diagnose lower extremity deep venous thrombosis (DVT). Our secondary end points include association of thromboembolic events with laboratory results, inflammatory markers and mortality.

## Materials and Methods

### Study Design and Definitions

We conducted a prospective observational study where all consecutive adult patients (≥18 years) with the laboratory confirmed diagnosis of Covid-19 admitted to the 8 ICUs across 3 hospitals of Montefiore Medical Center were included. Patients with previously diagnosed DVT or pulmonary embolism were excluded. The inclusion period was 35 days (4/22/2020-5/26/2020). We captured data through 6/9/2020 to provide 14 day follow up for all admitted patients. Our study was approved by the Institutional Review Board of the Albert Einstein College of Medicine. Thromboembolic events observed during the inclusion period were defined as deep or superficial venous thrombosis of upper or lower extremities, acute pulmonary embolism, acute ischemic stroke, acute myocardial infarction, arterial thrombosis and thrombosis at other sites. POCUS was used to screen and diagnose lower extremity DVT. Rest of the thromboembolic events were diagnosed by clinical criteria and standard imaging tests.

### POCUS Screening Examination

The Point of Care Ultrasound (POCUS) screening exam was performed at bedside by the research team to evaluate for deep venous thrombosis in bilateral lower extremities using “2- region technique” and assess overall cardiac function using basic critical care echocardiography.^30,31^ “2-region technique” involves compression of 1-2 cm area proximal and distal to the greater saphenous vein junction with the common femoral vein extending to the confluence of superficial and deep femoral vein and second area behind the knee extending from the proximal popliteal vein to the confluence of the calf vein. The examination was performed at 3 time points: within 24 hours of ICU admission (day 1), at day 7 and day 14 after the first exam. The upper extremity exam was performed if necessitated by the clinical suspicion of thrombosis such as swelling, redness or pain in upper extremities. Exams were performed on the wards, by the research team at the designated follow up time, for patients transferred out of ICU. No follow up exams were performed if patients expired or were discharged alive from the hospital. All positive POCUS exams were followed by an official ultrasound except for 3 patients that expired before the ultrasound was performed. All members of the research team (critical care fellows) routinely perform basic critical care echocardiography and deep venous thrombosis exams using POCUS as part of the management of critically ill patients. They are proficient in the performance of these bedside POCUS studies, having performed more than 150 each of those exams so far. All ultrasound images were archived in a picture archiving and communication system (Qpath E, Telexy) and were reviewed by 2 critical care medicine faculty who are experts in critical care ultrasonography (ALS, SK). For any discrepancies in POCUS and official ultrasound results, the images were carefully reviewed by the expert faculty and a final determination made. Our study ended on 6/9/2020 when we completed 14 day follow up exam for all remaining admitted patients. Patient demographics, co-morbidities, other thrombotic events and laboratory biomarkers were retrospectively collected after the completion of the inclusion period.

### Statistical Analysis

Descriptive statistics were generated, using means and standard deviations (SD) or medians and interquartile ranges (IQR) to summarize continuous variables, and frequencies (%) for categorical data. Characteristics of patients who developed thrombosis were compared to patients who did not develop thrombosis via independent t-tests or their non-parametric analog or chi-square tests. A Cox proportional hazards model for mortality was estimated to examine whether thrombosis is associated with a higher risk of death, treating the occurrence of thrombosis as a time-varying indicator variable. To examine the cumulative incidence of thrombosis after hospital admission, we used a non-parametric estimate of the cumulative incidence function (CIF), accounting for the competing risk of death. Secondary analyses examined cumulative incidence curves for specific types of venous thromboses (upper and lower), again treating death as a competing risk. All analyses were preformed using SAS version 9.4 (Copyright 2016; SAS Institute Inc., Cary, NC, USA).

## Results

### Demographics and Baseline Characteristics

A total of 107 patients were included in the study. The mean (+-SD) age was 60 (+-14) years and 62 (58%) were males. Fifty-nine (55%) of our patients were Hispanic and 32 (30%) were African-american. Hypertension was the most common co-morbidity seen in 74 (69%) patients, followed by diabetes mellitus in 48 (45%) and chronic kidney disease (CKD) in 21 (19.6%) patients. Mean body mass index was 29.7 kg/m2 and 12 patients (11%) had a prior history of venous thromboembolism.18 (17%) patients were taking aspirin or other anti-platelet agents prior to admission. Table 1 lists the baseline characteristics of the study population. Of 107 patients in the study, 44 were discharged alive, 50 died and 13 were still admitted in hospital at the end of study period (6/9/2020). The median ICU and hospital LOS were 7 days (IQR: 2-14 days) and 17 days (IQR: 9-28 days) respectively.

**Table 1.** Baseline Characteristics of Covid-19 Patients.

	Overall (n = 107)	Patients with any thromboembolic event (n = 40)	Patients without any thromboembolic event (n = 67)	P-value
Age- years Mean (SD)	60.3 (14.6)	64.3 (13.2)	57.9 (15.0)	.03
Male Gender- n (%)	62 (58.5)	20 (50.0)	42 (63.6)	.22
Race- n (%)				.04
African-American	32 (29.9)	17 (42.5)	15 (22.3)	
Hispanic	59 (55.1)	16 (40.0)	43 (64.2)	
White	10 (9.4)	5 (12.5)	5 (7.5)	
Other	6 (4.7)	2 (5.0)	4 (6.0)	
Co-morbidities- n (%)				
DM	48 (44.9)	17 (42.5)	31 (46.3)	.84
HTN	74 (69.2)	27 (70.2)	47 (67.5)	.83
CKD	21 (19.6)	6 (15.0)	15 (22.4)	.45
ESRD	7 (6.5)	1 (2.5)	6 (9.0)	.25
CAD	16 (15.0)	4 (10.0)	12 (17.9)	.40
CHF	12 (11.2)	2 (5.0)	10 (14.9)	.20
A.fib	8 (7.5)	1 (2.5)	7 (10.5)	.25
COPD	12 (11.2)	4 (10.0)	9 (13.4)	.53
Cancer	11 (10.3)	3 (7.5)	8 (11.9)	.53
HIV	2 (1.9)	0 (0.0)	2 (3.0)	.53
Cirrhosis	2 (1.9)	1 (2.5)	1 (1.4)	1.0
Admission origin- n (%)				.25
ED	43 (40.6)	16 (40.0)	27 (40.9)	
Floor	51 (48.1)	22 (55.0)	29 (43.9)	
Outside hospital	12 (11.3)	2 (5.0)	10 (15.2)	
Smoking status- n (%)				.11
Current	18 (16.8)	3 (7.5)	15 (22.4)	
Former	19 (17.6)	9 (22.5)	10 (14.9)	
Non-smoker	70 (65.4)	28 (70.0)	42 (62.7)	
Alcohol use- n (%)	26 (24.3)	6 (15.0)	20 (29.9)	.10
Drug abuse- n (%)	7 (6.5)	1 (2.5)	6 (9.0)	.25
BMI (kg/m^2^) Mean (SD)	29.7 (6.8)	30.6 (8.1)	29.2 (5.8)	.30
Prior Stroke- n (%)	12 (11.2)	4 (10.0)	8 (11.9)	1.0
H/o VTE- n (%)	12 (11.2)	5 (12.5)	7 (10.5)	.76
Use of Aspirin/anti platelets at home- n (%)	18 (16.8)	6 (15.0)	12 (17.9)	.79
DVT prophylaxis at the time of hospital admission- n (%)	96 (89.7%)	39 (97.5)	57 (89.1)	.05

Data are summarized as *mean(SD) or n (%)*, where n = available sample size.

Abbreviations: DM = Diabetes Mellitus, HTN = Hypertension, CKD = Chronic Kidney Disease, ESRD = End Stage Renal Disease, CAD = Coronary Artery Disease, CHF = Congestive Heart Failure, A.fib = Atrial Fibrillation, COPD = Chronic Obstructive Pulmonary Disease, HIV = Human Immunodeficiency Virus, ED = Emergency department, BMI = Body Mass Index, VTE = Venous Thromboembolism.

### POCUS Exam Results for Lower Extremity DVT Diagnosis

All patients got POCUS exam on day 1 in the ICU, 67 (62%) got day 7 follow up and 44 (41%) got day 14 follow up exam (Table 2). POCUS diagnosed 17 (15.9%) confirmed lower extremity DVTs on day 1, 3 (6%) new on day 7 and 1 (4.1%) new on day 14. Official ultrasound was performed in 64 patients overall and in 18 out of 21 patients with positive POCUS for lower extremity DVT. Discrepancies between POCUS and official ultrasound results were seen in 6 patients (Table 3), 4 of them were true positives, 1 false positive and 1 false negative by POCUS.

**Table 2.** POCUS Exam for Lower Extremity DVT Diagnosis.

	Day 1	Day 7	Day 14
Number of patients who got POCUS exam for LE DVT (n)	107	67	44
New Confirmed Positive LE DVT-n (%) % = patients with positive LE DVT/patients with no DVT diagnosed before	17 (15.9%)	3 (6.0%)	1 (4.1%)

Data are summarized as *n (%)*, where n = available sample size.

Abbreviations: POCUS = Point of Care Ultrasound, LE DVT = Lower extremity deep venous thrombosis.

**Table 3.** Discrepancy of the POCUS and Ultrasound Findings.

POCUS/ Ultrasound Discrepancies	Description	Final Inference by Faculty	Number of DVT exams (n)
True positive by POCUS	Official ultrasound did not confirm LE DVT diagnosed on POCUS, since there was a delay (average >72 hours) in the performance of official ultrasound. DVT was clearly visible on POCUS. Clot probably migrated before official ultrasound was performed.	Positive	4
False positive by POCUS	POCUS exam was incorrect as there was no DVT seen on official ultrasound performed on the same day.	Negative	1
False negative by POCUS	POCUS exam missed the DVT seen on official ultrasound exam.	Positive	1

Data are summarized as *n*, where n = available sample size.

Abbreviations: POCUS = Point of Care Ultrasound, LE DVT = Lower extremity deep venous thrombosis.

### Thromboembolic Events

Forty patients developed 52 thromboembolic events. Median (IQR) hospital day when thromboembolic event occurred was 5.5 days (2-18). Table 4 lists all observed thromboembolic events. Twenty-one lower extremity DVTs and 9 upper extremity thrombosis (5 DVT, 4 superficial venous thrombosis (SVT)) were diagnosed. Thirteen out of 21 lower extremity DVTs were proximal (at or above the knee) in location, 6 were distal (below knee) and 2 were extensive extending from groin to below the knee. Seventeen (81%) of the lower extremity DVTs were diagnosed on day 1 of ICU stay. Thirty patients underwent CT imaging to diagnose acute pulmonary embolism, of which, 13 were positive. Acute ischemic stroke developed in 4 patients and 4 thrombotic events were diagnosed at other sites (inferior vena cava, right ventricle, portal vein thrombosis). Ninety-six (90%) patients received routine deep venous thrombosis (DVT) prophylaxis at the time of hospital admission in the form of subcutaneous heparin, low molecular weight heparin (enoxaparin) or direct thrombin inhibitor (low dose apixaban).

**Table 4.** Thromboembolic Events (n = 52) (Total Number of Patients in Study = 107) Patients With Thromboembolic Events = 40.

Confirmed Lower Extremity DVT- (n)	
Total	21
Diagnosed on Day 1	17
Diagnosed on Day 7 (new)	3
Diagnosed on day 14 (new)	1
Site of Lower Extremity DVT- (n)	
Proximal	13
Distal Both	6 2
Upper Extremity- (n)	
Total	9
DVT	5
SVT	4
Acute pulmonary embolism (PE) diagnosed by CT Scan- (n)	13
Proximal/central	2
Distal/ segmental	11
Acute PE with RV thrombus	2/13
Acute ischemic stroke- (n)	4
Acute coronary syndrome/Myocardial Infarction- (n)	0
Arterial Thrombosis- (n)	1
Thrombosis at other sites- (n)	4
RV Thrombus without PE	1
IVC Thrombus	2
Portal vein Thrombus	1

Data are summarized as *n*, where n = available sample size.

Abbreviations: DVT = Deep venous thrombosis, SVT = Superficial venous thrombosis, RV = Right ventricular, IVC = Inferior vena cava.

### Laboratory Results and Outcomes

**Figure 1.**
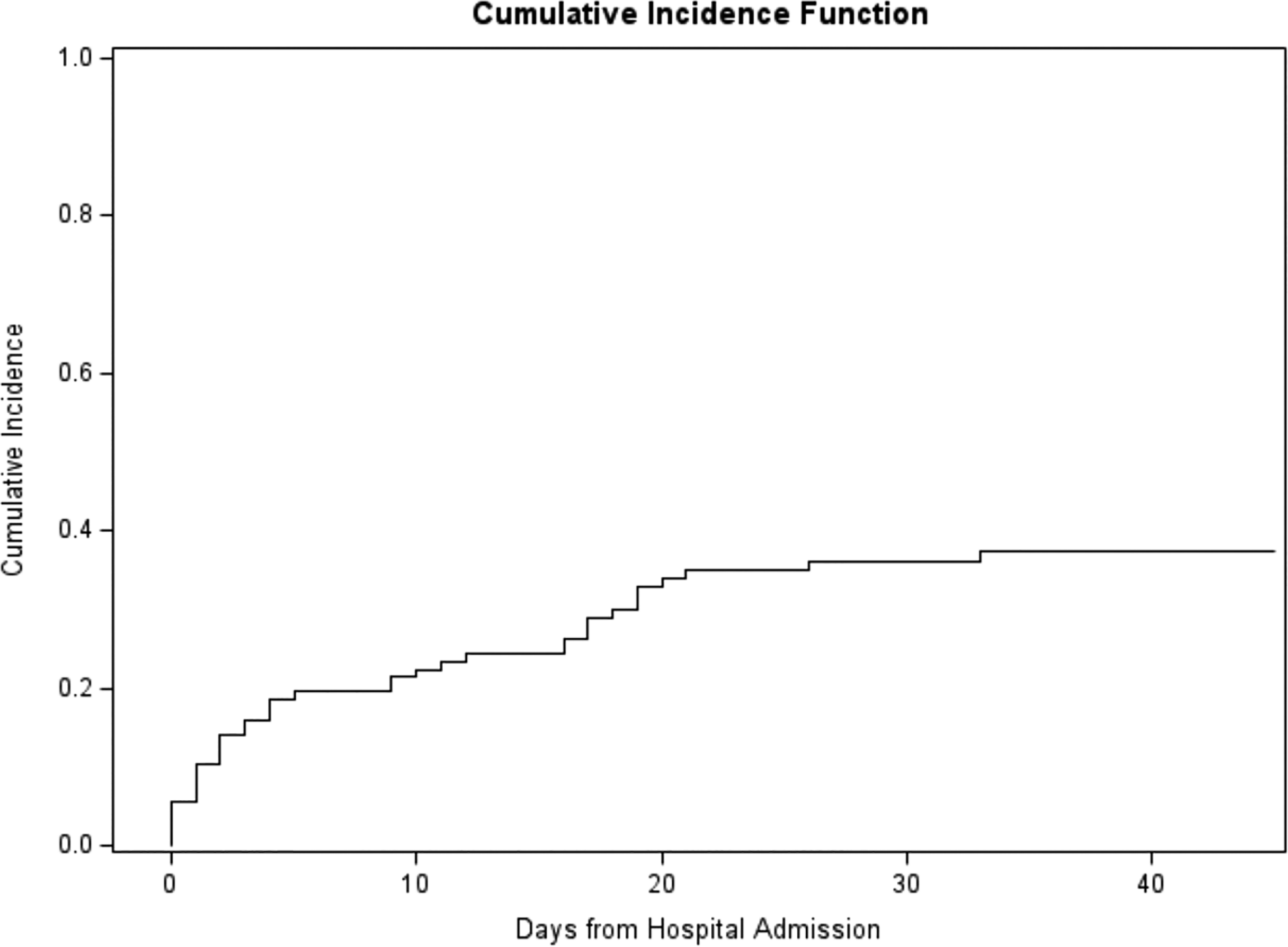
Cumulative incidence of thromboembolic event from hospital admission.

**Figure 2.**
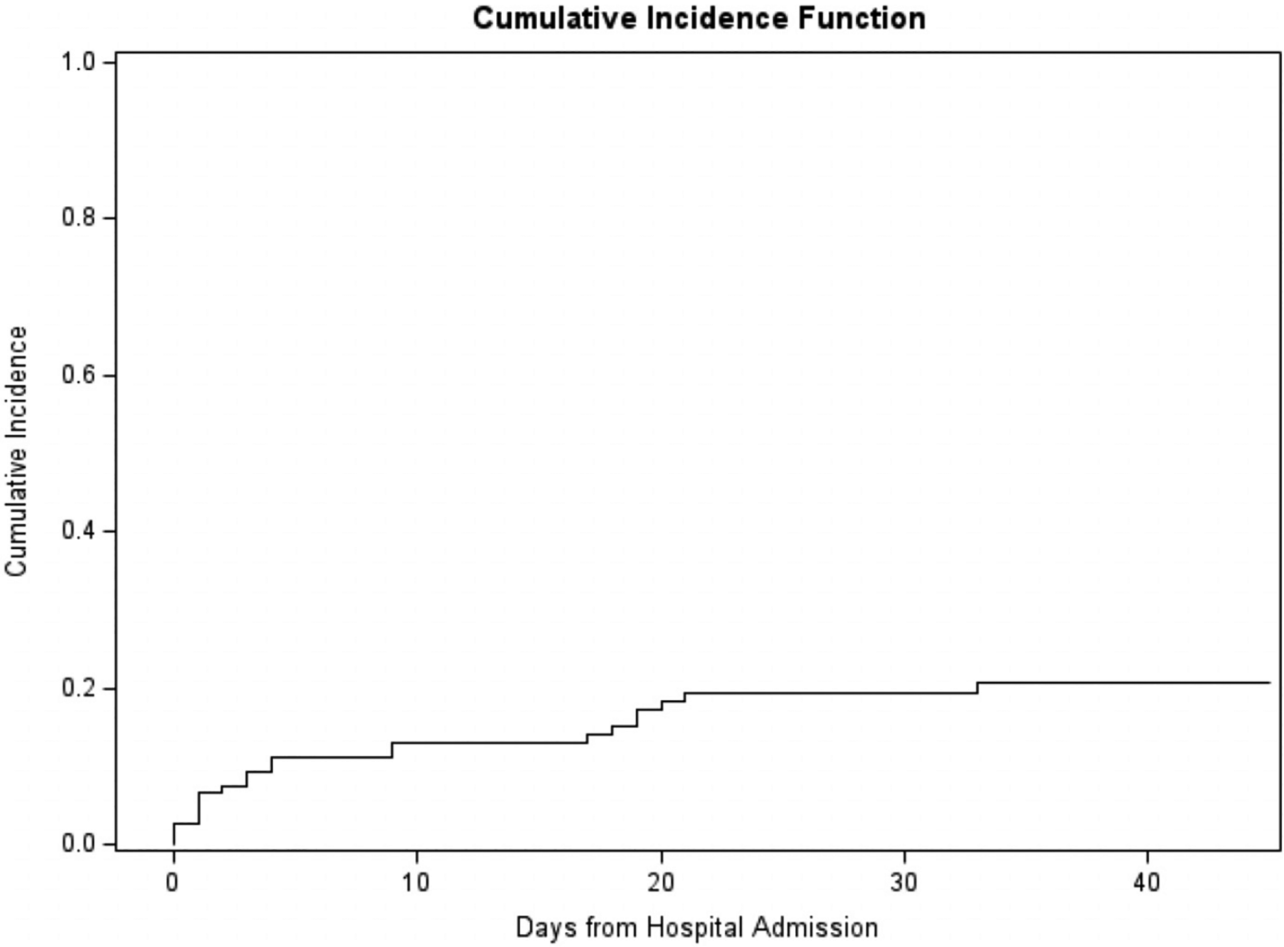
Cumulative incidence of lower extremity deep venous thrombosis from hospital admission.

**Figure 3.**
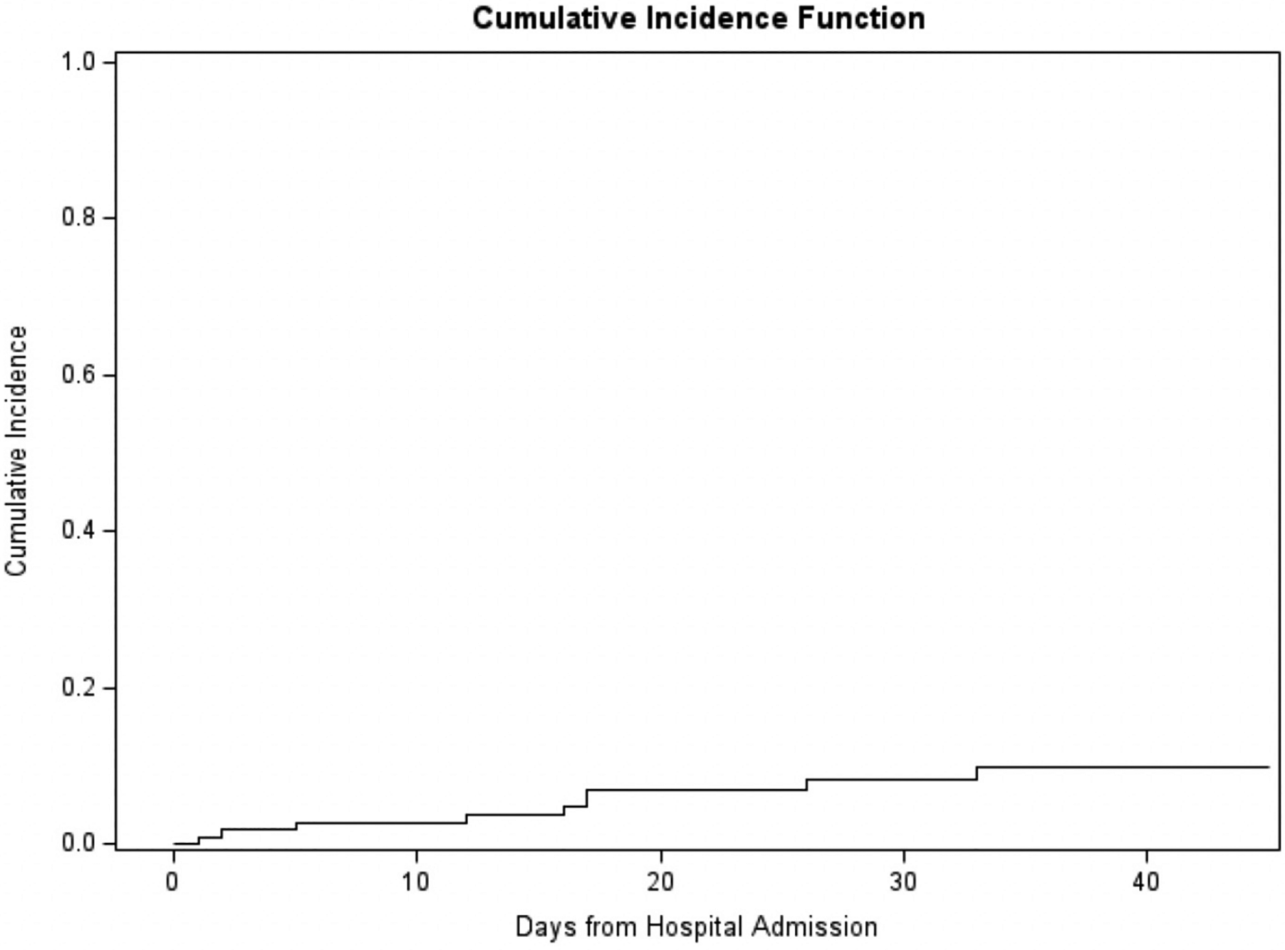
Cumulative incidence of upper extremity deep venous thrombosis from hospital admission.

Laboratory results of patients with and without thromboembolic event are presented in Table 5. Patients with thromboembolic events had statistically significant higher average peak d-dimer levels (15.0 versus 10.8; p-value = .01) compared to patients without any thromboembolic events. We did not observe any significant differences in other laboratory values and peak levels of inflammatory markers (ferritin, CRP, procalcitonin, fibrinogen) between patients with and without thromboembolic events. Thirteen (32.5%) patients had failure of therapeutic anticoagulation since they developed a thromboembolic event despite being on full dose anticoagulation. The cumulative incidence of any thromboembolic event was estimated to be 36% with a 95% CI of (27%, 45%) at 30 days post hospital admission and 37% with a 95% CI of (28%, 47%) at 45 days post hospital admission (Figure 1). At 45 days post hospitalization, the cumulative incidence of lower extremity DVT was estimated to be 21% with a 95% CI of (13%, 29%) and upper extremity DVT was estimated to be 10% with a 95% CI of (5%, 17%) (Figures 2 and 3). We also found that occurrence of a thromboembolic event was not associated with higher risk of mortality among Covid-19 patients in ICU (HR of 1.08, p value = .81).

**Table 5.** Laboratory Results in Covid-19 Patients.

	Overall	Patients with any thrombotic event	Patients with no thrombotic event	P-value*
D-dimer admission (microgram/ml)	5.6(6.5); [0.3-20]; n = 100	6.7(7); [0.5-20]; n = 37	5(6.1); [0.3-20]; n = 63	0.22
D-dimer peak^^^ (microgram/ml)	12.4(7.7); [0.3-26.9]; n = 106	15.0(6.8); [2-20]; n = 40	10.8(7.8); [0.3-26.9]; n = 66	0.01
D-dimer nadir (microgram/ml)	2.7(3.1); [0.3-19.9]; n = 106	2.7(2.4); [0.5-12.2]; n = 40	2.7(3.5); [0.3-19.9]; n = 66	0.92
Platelet count admission (K/microliter)	265.6(163.3); [43-1477]; n = 107	242.9(95.7); [103-516]; n = 40	279.2(192.1); [43-1477]; n = 67	0.27
Platelet count nadir (K/microliter)	164.1(102.8); [13-840]; n = 107	163.4(64.3); [69-433]; n = 40	164.5(120.5); [13-840]; n = 67	0.96
Fibrinogen peak (mg/dl)	707.4(228.6); [145-1251]; n = 96	687.4(211.7); [258-1176]; n = 37	719.8(239.5); [145-1251]; n = 59	0.50
Fibrinogen nadir (mg/dl)	474.4(207.6); [30-964]; n = 96	437.8(195.3); [125-808]; n = 37	497.3(213.3); [30-964]; n = 59	0.17
INR admission	1.4(1); [0.9-7.3]; n = 105	1.5(1.2); [1-7.3]; n = 38	1.4(0.8); [0.9-7]; n = 67	0.37
INR peak	2.2(1.9); [1-14.4]; n = 107	2.2(1.6); [1.1-9.4]; n = 40	2.2(2.1); [1-14.4]; n = 67	0.99
Ferritin peak (ng/ml)	7388.3(18533.6); [54-100000]; n = 105	9050.4(23229.6); [321-100000]; n = 39	6406.1(15214.7); [54-97915]; n = 66	0.48
C-RP peak (mg/dl)	25.8(14.3); [0.5-75.6]; n = 105	27.6(15.6); [0.5-75.6]; n = 40	24.7(13.5); [0.5-52.5]; n = 65	0.30
Procalcitonin peak (ng/ml)	7.5(12.5); [0.1-50]; n = 99	5.5(9.9); [0.1-42.3]; n = 37	8.8(13.7); [0.1-50]; n = 62	0.20

Data are summarized as *mean(SD); [min-max]; n*, where n = available sample size.

* Corresponding to a 2-sided t-test for 2 independent groups. Note: results based on non-parametric analog are qualitatively identical.

^ Corresponds to peak values during entire hospitalization.

Abbreviations: INR = International normalized ratio, CRP = C-reactive protein, ICU = Intensive care unit.

## Discussion

Our study reports high rates (37.3%) of thromboembolic events in the Covid-19 ICU population with high cumulative incidence of thromboembolism of 37% at 45 days. There was also a high 45-day cumulative incidence of lower and upper extremity DVT of 21% and 10% respectively. Thus, critically ill Covid-19 patients have a much higher risk of developing thromboembolic event compared to general ICU population whose rates are estimated at 5-10%.^32,33^ Our findings are similar to prior studies reporting rates of thromboembolic events ranging from 18% to 69% in Covid-19 patients in ICU.^10,12,15–19,21–26^ Lower extremity DVT rates in ICU Covid-19 patients show wide variability with values as low as 2% or less to as high as 23% to 54% per multiple reports.^10,12,15–19,22–26^ Klok et al reported 49% cumulative incidence of thromboembolic complications, of which, only 3 were lower extremity DVTs.^15^ Lodigiani reporting the Italian experience on 61 ICU patients also found only 3 lower extremity DVTs.^16^ A French study by Helms et al on 150 ICU patients revealed a thromboembolic complication rate of 18% but with only 2% as isolated leg DVT.^12^ Our findings report higher cumulative incidence of lower extremity DVTs detected by serial lower extremity POCUS exams at scheduled time intervals compared to other studies where selection bias was present and DVTs were diagnosed only if ultrasound imaging was ordered. Voicu and colleagues performed a prospective study where they performed duplex ultrasonography on 56 mechanically ventilated Covid-19 patients and found a high incidence of lower extremity DVT of 46%.^24^ They performed initial ultrasound at 3 days after intubation and second ultrasound at 8 days after intubation if initial ultrasound was negative. Seventy-seven (77%) of their DVTs were diagnosed on day 3 after intubation. This is similar to our findings where we found majority of our lower extremity DVTs (81%) at day 1 of ICU admission.

POCUS is an excellent tool utilized by Intensivists and helps make important diagnostic and management decisions.^34,35^ It represents an attractive option during the Covid-19 pandemic since it is portable technology, can be performed readily by clinicians at bedside, is reproducible, reduces exposure of additional personnel and avoids transmission of infection by avoiding patient transport. The sensitivity of limited bedside lower extremity ultrasound for the diagnosis of deep vein thrombosis varies from 84% to 97% and specificity of >95%.^36,37^ The use of POCUS in Covid-19 patients is also endorsed and encouraged by The American College of Chest Physicians (ACCP) expert panel and American College of Emergency Physicians.^35,38^ ACCP suggested to use lower extremity ultrasound as part of POCUS screening in Covid-19 patients with suspected pulmonary embolism, unexplained right ventricular dysfunction or unexplained refractory hypoxemia. Tavazzi et al in their report strongly suggested a close vein ultrasound screening and monitoring to be performed in all Covid-19 patients to screen for DVTs.^39^

We did notice discrepancy in the findings of POCUS and official ultrasound in 6 patients, 4 of them were true positives, 1 false positive and 1 false negative by POCUS.

Our faculty confirmed these findings by carefully reviewing images in the Qpath storage software and comparing them to official ultrasound results. We diagnosed DVT in 4 patients with POCUS when official ultrasound was negative because the ultrasound was performed on an average delay of more than 72 hours after the initial POCUS. The clot probably migrated by the time ultrasound was performed since 2 of those patients had confirmed acute pulmonary embolism.

Our study found higher peak d-dimer levels in patients with thromboembolic complications compared to patients with none. Elevated d-dimer level is very frequently observed in Covid associated coagulopathy and higher levels correlate with more severe disease and in-hospital mortality^40^. Tang et al reported higher d-dimer levels in non survivors compared to survivors and that anticoagulant treatment is associated with decreased mortality^41,42^ We did observe a high failure rate of DVT prophylaxis therapy and therapeutic anticoagulation in our patients with thromboembolism. This has been confirmed in a recent study by Maatman et al where 93% patients were on chemoprophylaxis or full dose anticoagulation and developed venous thromboembolic (VTE) events.^18^ Llitjos et al reported 56% VTE and 6 pulmonary embolisms in patients on therapeutic anticoagulation.^17^ Future studies are warranted to study the intensity of anticoagulation in this population to ensure more effective prevention.

Klok and colleagues reported that patients with thrombotic complications were at higher risk of all cause death with hazard ratio of 5.4.^15^ This is in contrast to our findings where we did not observe increased hazard of death between patients with and without thromboembolic events. Even though systemic inflammation is implicated in the pathogenesis of Covid-19 associated coagulopathy, we did not see a statistically significant difference in the peak levels of inflammatory markers in patients who developed thromboembolic events compared to patients with none.

The major strengths of our study are its prospective nature with14-day follow up available in 41% of patients. Another strength is the use of POCUS to screen for lower extremity thrombosis which helped us diagnose many asymptomatic DVTs earlier. Limitations of our study include single center study design, less aggressive use of CT scans to diagnose pulmonary embolism compared to other studies and that 12% of the patients did not have definite outcome as they were still admitted in the hospital at the end of follow up period. Also, official ultrasound was performed in only 64 (60%) of our study patients.

## Conclusions

Covid-19 patients in ICU are at increased risk of developing thromboembolic complications. We report a high cumulative incidence of overall thromboembolic events, including lower and upper extremity DVTs. Thromboembolic events were not associated with a higher risk of mortality in our cohort. There was a detectable failure rate of therapeutic anticoagulation in this population. POCUS is an excellent and feasible option to help screen for and diagnose thromboembolic events during a pandemic with limited availability of resources.
